# Increased amino acids levels and the risk of developing of hypertriglyceridemia in a 7-year follow-up

**DOI:** 10.1007/s40618-013-0044-7

**Published:** 2014-01-09

**Authors:** D. O. Mook-Kanamori, W. Römisch-Margl, G. Kastenmüller, C. Prehn, A. K. Petersen, T. Illig, C. Gieger, R. Wang-Sattler, C. Meisinger, A. Peters, J. Adamski, K. Suhre

**Affiliations:** 1 Department of Physiology and Biophysics, Weill Cornell Medical College, Qatar, PO Box 24144 Doha, Qatar; 2 Department of Endocrinology and Metabolic Diseases, Leiden University Medical Centre, Leiden, The Netherlands; 3 Helmholtz Zentrum München, German Research Center for Environmental Health, Institute of Bioinformatics and Systems Biology, Neuherberg, Germany; 4 Genome Analysis Center, Helmholtz Zentrum München, German Research Center for Environmental Health, Institute of Experimental Genetics, Neuherberg, Germany; 5 Research Unit of Molecular Epidemiology I, Helmholtz Zentrum München, German Research Center for Environmental Health, Neuherberg, Germany; 6 Hannover Unified Biobank, Hannover Medical School, Hannover, Germany; 7 Helmholtz Zentrum München, German Research Center for Environmental Health, Institute of Genetic Epidemiology, Neuherberg, Germany; 8 Helmholtz Zentrum München, German Research Center for Environmental Health, Institute of Epidemiology II, Neuherberg, Germany; 9 Technische Universität München, Munich, Germany

**Keywords:** Amino acids, Hypertriglyceridemia

## Abstract

**Background:**

Recently, five branched-chain and aromatic amino acids were shown to be associated with the risk of developing type 2 diabetes (T2D).

**Aim:**

We set out to examine whether amino acids are also associated with the development of hypertriglyceridemia.

**Materials and methods:**

We determined the serum amino acids concentrations of 1,125 individuals of the KORA S4 baseline study, for which follow-up data were available also at the KORA F4 7 years later. After exclusion for hypertriglyceridemia (defined as having a fasting triglyceride level above 1.70 mmol/L) and diabetes at baseline, 755 subjects remained for analyses.

**Results:**

Increased levels of leucine, arginine, valine, proline, phenylalanine, isoleucine and lysine were significantly associated with an increased risk of hypertriglyceridemia. These associations remained significant when restricting to those individuals who did not develop T2D in the 7-year follow-up. The increase per standard deviation of amino acid level was between 26 and 40 %.

**Conclusions:**

Seven amino acids were associated with an increased risk of developing hypertriglyceridemia after 7 years. Further studies are necessary to elucidate the complex role of these amino acids in the pathogenesis of metabolic disorders.

## Introduction

Hypertriglyceridemia is one of the most important risk factors for cardiovascular diseases, such as myocardial infarction and stroke [1]. Over the past decade studies have focused on searching for biomarkers that would allow for early prediction of hypertriglyceridemia. Genome-wide association studies have revealed numerous loci that are associated with triglyceride levels [2]. However, the overall explained proportion of the heritability by the gene variants is low (about 10 %) and the predictive value is limited [3].

Mass spectroscopy allows for the simultaneous measurement of many metabolites from a single sample and has proven to be a powerful and accurate tool for biomarker research [4–7]. A recent genome-wide association study showed strong association between several loci of genetic variance and metabolite levels [4]. Metabolites also serve as intermediate traits in the analysis of gene–environment interactions and the development of complex diseases, such as type 2 diabetes (T2D) and cardiovascular disorders [4]. Metabolic traits are functional readouts of biological processes and may improve disease prediction when they are close to a pathogenic pathway [4]. Most interesting are longitudinal studies that associate metabolic patterns at baseline with clinical outcomes in a follow-up study that is ideally conducted many years later. However, few longitudinal studies with metabolic traits measured at baseline are available so far [7].

One successful example of a study identifying metabolic biomarkers of longitudinal disease outcome is the Framingham Offspring Study. Wang et al. [7] found that five branched-chain and aromatic amino acids were associated with the risk of developing T2D in a 12-year follow-up. These findings have been replicated in other follow-up studies [8, 9]. Increased amino acid levels have been shown to induce skeletal muscle insulin resistance [10]. Also, branched-chain and aromatic amino acid levels are known to be increased in obese individuals [11]. Branched-chain amino acids have also been suggested to have a role in pathophysiological processes in heart disease [12]. Thus, there appears to be a relationship between these metabolic biomarkers and the pathogenesis of cardio-metabolic diseases. However, the precise role of these amino acids in the development of dyslipidemia remains largely unknown.

Since hypertriglyceridemia is an important independent cardiovascular risk factor, we ask the question of whether amino acids are associated with hypertriglyceridemia, independent of diabetes, and examine the relationship between amino acids and hypertriglyceridemia in a 7-year follow-up in a population-based study. We hypothesized that especially baseline branched-chain and aromatic amino acid levels would be associated with hypertriglyceridemia.

## Research design and methods

### Study design and population

This study was embedded in the KORA S4 study (S4) and its 7-year follow-up KORA F4 study (F4). KORA (Cooperative Health Research in the Region of Augsburg) is a research platform that has been performing longitudinal population-based surveys and sampling and has been described previously in detail [13, 14]. This study was approved by the ethics committee of the Bavarian Medical Association. Written informed consent was obtained from all participants in accordance with institutional guidelines and the Declaration of Helsinki Principles.

The amino acid levels of 1,614 subjects aged 55–74 years in S4 were determined. Fasting triglyceride, amino acids levels, and information regarding T2D at both time points were available in 1,125 subjects. Hypertriglyceridemia was defined as having a fasting triglyceride level above 1.70 mmol/L [1]. T2D was defined as being diagnosed as a person with T2D based on a self-reported physician diagnosis and consequently validated by contacting the treating physician. After exclusion of subjects with hypertriglyceridemia and T2D at baseline, 755 subjects were eligible for analyses.

### Amino acid and triglyceride measurements

In both S4 and F4, fasting serum samples (at least 8 h) for amino acids and triglycerides were collected during the study center visit and has been described previously [14, 15]. Targeted metabolite quantification by electrospray ionization (ESI) tandem mass spectrometry (MS/MS) was performed using the AbsoluteIDQ™ kits p180 (BIOCRATES Life Sciences AG, Austria) for the KORA surveys S4 and resulting in the quantification of 188 metabolites. The techniques using LC–MS/MS and/or FIA-MS/MS (FIA: flow injection analysis) have previously been described in detail [15, 16]. Briefly, the assay preparation was done by an automated robotics system (Hamilton Bonaduz AG, Switzerland) on a special 96-well sandwich plate which implemented filter containing stable isotope-labeled internal standards. Assays used 10 μL plasma and included phenylisothiocyanate (PITC) derivatisation of amino acids, extraction with organic solvents and several liquid handling steps. In S4, 21 amino acids (19 proteinogenic + citrulline + ornithine) have been quantified. The quantification of the metabolites is achieved by reference to appropriate internal standards. The method is proven to be in conformance with the “guidance for industry—bioanalytical method validation” published by the food and drug administration (FDA), which implies proof of reproducibility within a given error range [17]. Fasting triglycerides (TG) were measured with the TGL Flex GPO-PAP assay (Dade Behring, Germany).

### Covariates

Weight and height were measured to the nearest 0.1 kg and 0.1 cm, respectively. Body mass index (BMI) was calculated as (weight/height^2^). Age and diagnosis of T2D were obtained through questionnaire. Self-reported physician-diagnosed T2D state was validated by contacting the treating physician.

### Statistical analyses

First, for each of the 21 amino acids multivariate logistic regression was performed to assess the risk of developing hypertriglyceridemia after 7 years, adjusting for age, gender, and body mass index (model 1). Subsequently, these analyses were additionally adjusted for baseline triglyceride levels (model 2). Then, model 2 was additionally restricted to subjects without T2D for both in S4 and F4 (model 3). Finally, to account for the possible residual confounding of pre-diabetes or insulin resistance, model 3 was additionally adjusted for hexose levels, which has been shown by the manufacturer to contain over 90 % glucose (model 4). Second, we examined the relationship between the significantly associated amino acids and the risk of developing hypertriglyceridemia further using odds ratios per standard deviation increase of amino acid level and quartiles. Third, we examined the predictive value of the amino acids using the area under the curve (AUC) on a ROC-curve. Restricting to non-diabetics, we first determined the AUC for the risk of developing hypertriglyceridemia using age, gender and BMI alone. Subsequently, we examined whether adding one amino acid at a time significantly increased the AUC. No correction for multiple testing was applied since many of the amino acids are highly correlated to each other. All statistical analyses were performed in Statistical Package for Social Sciences version 20.0 (SPSS Inc, Chicago, IL, USA).

## Results

Subject characteristics are shown in Table 1. The median follow-up time was 7 years (90 % range 6–8 years). Table 2 shows the* p* values for logistic regression analyses for the 21 amino acids on hypertriglyceridemia after 7-year follow-up. After adjustment for gender, age and body mass index, seven amino acids were significantly associated with hypertriglyceridemia (leucine, arginine, valine, proline, phenylalanine, isoleucine and lysine). These associations remained significant after adjustment for baseline triglyceride levels and restriction to non-diabetic subjects. However, after additional adjustment for serum hexose, isoleucine (*p* = 0.054) and lysine (*p* = 0.069) were no longer statistically significant.

**Table 1:** Subject characteristics (*n* = 755)

Subject characteristic	Baseline	7-year follow-up
Age (years)	63 (55–72)	70 (62–80)
Gender (% male)	399 (52.8 %)	399 (52.8 %)
Weight (kg)	75.2 (56.6–97.6)	75.5 (56.1–98.5)
Height (cm)	165.0 (8.7)	164.7 (8.8)
Body mass index (kg/m^2^)	27.4 (21.8–35.5)	27.5 (21.5–36.0)
Triglyceride level (mmol/L)	1.10 (0.59–1.60)	1.13 (0.59–2.30)

Values represent means (standard deviations), medians (90 % range), or number of subjects (%)

**Table 2:** Relation of baseline amino acid levels and the development of hypertriglyceridemia after 7 years

Amino acid levels at baseline	Model 1	Model 2	Model 3	Model 4
Arginine	**0.009**	**0.012**	**0.009**	**0.017**
Leucine	**0.001**	**0.022**	**0.010**	**0.018**
Valine	**8.2** **×** **10** ^**−4**^	**0.021**	**0.013**	**0.027**
Proline	**0.002**	**0.008**	**0.017**	**0.029**
Phenylalanine	**0.009**	0.057	**0.021**	**0.038**
Isoleucine	**9.4** **×** **10** ^**−4**^	**0.049**	**0.033**	0.054
Lysine	**0.022**	0.073	**0.042**	0.069
Aspartic acid	0.094	0.21	0.11	0.13
Serine	0.24	0.15	0.12	0.17
Ornithine	0.11	0.20	0.14	0.19
Glutamine	0.31	0.19	0.16	0.20
Tyrosine	0.10	0.39	0.21	0.28
Methionine	0.16	0.47	0.31	0.32
Histidine	0.41	0.67	0.41	0.50
Asparagine	0.91	0.51	0.48	0.50
Glutamic acid	0.071	0.34	0.33	0.54
Citrulline	0.58	0.55	0.71	0.54
Glycine	0.87	0.81	0.72	0.69
Threonine	0.86	0.71	0.73	0.69
Tryptophan	0.47	0.90	0.67	0.81
Alanine	0.60	0.72	0.70	0.84

*n* = 755 (121 cases) for model 1 + 2; *n* = 686 (103 cases) for model 3

Values represent *p* values for logistic regression analyses for the risk of developing hypertriglyceridemia (>1.70 mmol/L). All amino acid levels were log-transformed

Values in bold are significant at *p* < 0.05

Model 1: adjusted for gender, age and body mass index at baseline

Model 2: adjusted for gender, age, body mass index and triglyceride levels at baseline

Model 3: adjusted for gender, age, body mass index and triglyceride levels at baseline and restricted to non-diabetic subjects (both at baseline and at 7 years)

Model 4: adjusted for gender, age, body mass index, triglyceride and hexose levels at baseline and restricted to non-diabetic subjects (both at baseline and at 7 years)

Table 3 gives the odds ratio for these seven amino acids for the risk of developing hypertriglyceridemia. All seven amino acids showed an increase in risk of hypertriglyceridemia. The strongest effect was found for leucine, where the per standard deviation increase was 40 % (95 % confidence interval 9–80 %). When examining the odds ratios per quartile of amino acid level, lysine was the only amino acid that showed a significant trend increase (*p* = 0.05).

**Table 3:** Odds ratios for the risk of the development of hypertriglyceridemia after 7 years

	Leucine	Arginine	Valine	Proline	Phenylalanine	Isoleucine	Lysine
Amino acid as continuous variable							
Per SD	1.40 (1.09–1.80)	1.36 (1.08–1.72)	1.37 (1.07–1.75)	1.35 (1.05–1.72)	1.30 (1.04–1.64)	1.33 (1.02–1.73)	1.26 (1.01–1.59)
*p* value	**0.009**	**0.01**	**0.01**	**0.02**	**0.02**	**0.03**	**0.04**
Amino acid as categorical variable							
First quartile	1.0 (reference)	1.0 (reference)	1.0 (reference)	1.0 (reference)	1.0 (reference)	1.0 (reference)	1.0 (reference)
Second quartile	1.21 (0.57–2.54)	1.26 (0.65–2.45)	1.70 (0.82–3.55)	0.82 (0.39–1.82)	0.79 (0.39–1.63)	1.14 (0.54–2.38)	0.51 (0.25–1.05)
Third quartile	2.03 (1.00–4.12)	1.46 (0.76–2.82)	1.31 (0.62–2.79)	1.75 (0.89–3.42)	1.34 (0.70–2.58)	1.31 (0.62–2.77)	1.07 (0.57–2.02)
Fourth quartile	1.65 (0.79–3.43)	1.52 (0.80–2.91)	2.15 (1.05–4.42)	1.53 (0.76–3.07)	1.59 (0.84–3.00)	1.72 (0.81–3.66)	1.42 (0.76–2.61)
*p* for trend	0.13	0.18	0.06	0.06	0.06	0.12	0.05

*n* = 686 (103 cases). Analyses restricted to non-diabetic subjects (both at baseline and at 7 years)

Values represent odds ratios (95 % confidence intervals) for the risk of developing hypertriglyceridemia (>1.70 mmol/L)

Values in bold are significant at *p* < 0.05

Model is adjusted for gender, age, body mass index and triglyceride levels at baseline

The predictive value, measured by area under the curve (AUC) of a ROC-curve, for gender, age, body mass index and triglyceride level on the risk of hypertriglyceridemia was 72.3 %. Though all seven significantly associated amino acids did improve this model slightly, the improvement was minimal and not statistically significant (largest increase for proline was 1.7 %).

## Discussion

In this population-based study, we show associations of seven amino acids with the risk of developing hypertriglyceridemia after 7-year follow-up. All seven remained significant after restriction to non-diabetic subjects (both at baseline and follow-up) and five of them remained significant after adjustment for hexose. Several of the amino acids found to be associated with hypertriglyceridemia (more precisely, isoleucine, leucine, valine and phenylalanine), overlap with previous findings regarding the risk of T2D [7]. Nonetheless, the predictive value of these amino acids over conventional risk factors remains limited.

Some methodological issues should be considered. Subjects with metabolite data were significantly older than those without. We do not expect that this difference in age materially influenced our results, but we cannot exclude that the associations that we found may be limited to older age groups. We classified hypertriglyceridemia using a cut-off of 1.70 mmol/L, which is recommended by the American Heart Association [1]. Using other cut-offs did not materially change the results, making misclassification of hypertriglyceridemia unlikely (data not shown). Finally, Bonferroni correction for multiple testing would be overly conservative, since many of the amino acids are highly correlated and not independent determinants. Also, the a priori chance of amino acids being associated with hypertriglyceridemia is higher, due to their known role in glucose metabolism.

The relationship between amino acids, hypertriglyceridemia and T2D is complex. Hypertriglyceridemia together with both normal and impaired glucose levels predicts the development of T2D [18, 19]. Wang et al. [7] previously described that higher levels of isoleucine, leucine, valine, tyrosine and phenylalanine were associated with an increased risk of T2D after a 12-year follow-up. We found that these same amino acids (except tyrosine) were also associated with hypertriglyceridemia. The associations between amino acids and hypertriglyceridemia were found in non-diabetic subjects. Firstly, a potential mediating mechanism for these associations may be through the branched-chain amino acid-derived acylcarnitines, C3- and C5-acylcarnitines. Studies have shown that branched-chain and aromatic amino acids, as well as C3- and C5-acylcarnitines, can affect insulin sensitivity and markers of insulin resistance [20, 21]. In our study, C3-acylcarnitine was also associated with hypertriglyceridemia (*p* = 0.003), but C5-acylcarnitine (*p* = 0.16) was not (data not shown). Acylcarnitines are modified fatty acids (which are derived from triglycerides) that are transported through the mitochondrial membrane for beta-oxidation [22]. Thus, acylcarnitines also directly reflect the oxidation rate of amino acids and fatty acids, which may explain why we find associations between these amino acids and hypertriglyceridemia in subjects without T2D. Secondly, our finding that the associations between these amino acids and hypertriglyceridemia persisted after excluding patients with T2D does not mean that the mechanisms are unrelated to insulin sensitivity (which may be present among non-diabetic subjects). In fact, a mechanistic relationship between amino acids and insulin sensitivity, as suggested by the observations by Wang et al. [7], may be mediating the observations between these amino acids and the risk of developing T2D and hypertriglyceridemia. However, the fact that the additional statistical adjustment for hexose did not materially change the associations between amino acids and hypertriglyceridemia, make it less likely that these associations are mediated through an insulin-resistant state. A third potential explanation is that these amino acids may be markers for a more general and confounding metabolic phenotype such as body fat mass or dietary intake. Interestingly, already several decades ago a small study showed correlations of branched-chain and aromatic amino acids with obesity [11]. In the current study, adjustment for body mass index markedly decreased the effect size on hypertriglyceridemia. Nonetheless, the associations for seven amino acids remained significant, indicating that at least a part of the effect of amino acids on triglyceride levels is not mediated through body mass index.

Over the past years an abundance of studies have focused on biomarkers of metabolic diseases, such as hypertriglyceridemia and diabetes. Genome-wide association studies have led to the identification of several new biological pathways, such as *FADS1*, but all these genes can only explain up to 10 % of the variance in triglyceride levels [2]. Using a hypothesis-free approach to examine numerous metabolites by means of mass spectroscopy has proven to be successful in biomarker discovery [7]. These biomarkers could serve as intermediate between the genes and the phenotype, thus giving a better prediction [4]. However, in the current study the additive value of the amino acids was marginal over classical risk factors, such as body mass index. Also, a recent genome-wide association study on (amongst others) amino acids, did not reveal any associations with genes related to obesity, T2D or triglyceride levels, further underlining the complexity of the pathological mechanisms in these metabolic disorders [4].

In conclusion, this study identified seven amino acids that are associated with an increased risk of developing hypertriglyceridemia after 7 years. Further and longer follow-up studies are necessary to elucidate the complex role of these amino acids in the pathogenesis of the metabolic disorders.
